# Sentido de coherencia en mujeres lactantes: una revisión de alcance

**DOI:** 10.23938/ASSN.1064

**Published:** 2024-02-09

**Authors:** Ester Sierra-García, Carlos Saus-Ortega

**Affiliations:** 1 Hospital Universitari i Politècnic La Fé de València València España; 2 Escuela de Enfermería La Fé Universitat de València València España; 3 Grupo de Investigación GREIACC. Instituto de Investigación Sanitaria La Fé. València, España. Grupo de Investigación GREIACC Instituto de Investigación Sanitaria La Fé València España

**Keywords:** Sentido de coherencia, Lactancia materna, Salud materno infantil, Adaptación psicológica, Sense of coherence, Breast feeding, Maternal and Child Health, Adaptation, Psychological

## Abstract

**Fundamento:**

El establecimiento de la lactancia materna puede resultar una situación potencialmente estresante. El objetivo del estudio es analizar el sentido de coherencia en mujeres lactantes, establecer los recursos generales de resistencia que lo modulan, y determinar las intervenciones profesionales que lo promueven.

**Metodología:**

Se incluyeron estudios en inglés, español o portugués, que evaluaran el sentido de coherencia de las mujeres lactantes, localizados en las bases de datos *PubMed*, *PsycINFO*, *ScienceDirect* y CINAHL entre marzo y mayo de 2023. La calidad de los estudios y el riesgo de sesgo se evaluaron siguiendo los criterios ICROMS y STROBE.

**Resultados:**

Se identificaron 316 registros, de los que se incluyeron un total de ocho estudios, tres cualitativos y cinco cuantitativos, todos con calidad suficiente. Un alto nivel de sentido de coherencia materno se relacionó con mayor duración, autoeficacia y disfrute de la experiencia de lactancia, y mayor apego. Los principales recursos generales de resistencia fueron percibir apoyo social, especialmente de parejas, madres y personal sanitario, además de experiencia previa positiva y una actitud positiva. Las intervenciones que favorecieron el sentido de coherencia fueron las relacionadas con un apoyo profesional estrecho, empático, personalizado, integral y centrado en la familia.

**Conclusiones.:**

La determinación del nivel de sentido de coherencia en madres lactantes puede ayudar a identificar a mujeres con mayor riesgo de destete temprano, y a establecer estrategias de intervención profesional que mejoren la experiencia de lactancia materna.

## Abstract (en)

**Background:**

The establishment of breastfeeding may sometimes be stressful. We aimed to analyze the sense of coherence in lactating women to determine the general resistance resources during lactation and the professional interventions that promote a high sense of coherence.

**Methods:**

A search of studies in English, Spanish, or Portuguese on lactating women’s sense of coherence was carried out in PubMed, PsycINFO, ScienceDirect, and CINAH databases published between May and November 2022. Study quality and risk of bias were examined according to ICROMS and STROBE criteria.

**Results:**

We identified 316 studies, of which eight -all of adequate quality- were included, three qualitative and five quantitative. A high level of maternal sense of coherence was related to longer duration, self-efficacy, attachment, and enjoyment of the breastfeeding experience. The main general resistance resources were to receive social support, particularly from partners, mothers, and health professionals. The interventions that favored the sense of coherence were mainly those related to a close, empathic, personalized, comprehensive, and family-centered professional support.

**Conclusions:**

The detection of the level of sense of coherence in lactating mothers may help identify women with a higher risk of weaning and establish professional intervention strategies that improve the breastfeeding experience.

## INTRODUCCIÓN

La lactancia materna (LM) se asocia, en forma de dosis-respuesta, con menores tasas de morbimortalidad y mayores beneficios materno-infantiles^1^^-^^3^. A nivel infantil previene las diarreas, neumonías o la obesidad^4^; mientras que a nivel materno protege frente al cáncer de mama y ovario, reduce el estrés y los niveles de ansiedad y a su vez fortalece el vínculo madre-hijo^5^^-^^7^. A pesar de estos importantes beneficios, las tasas mundiales de lactancia materna exclusiva son del 48%^8^, cerca del objetivo del 50% recomendado por la OMS-UNICEF para 2025; sin embargo, se proyecta a nivel mundial alcanzar un 70% de tasa de lactancia materna exclusiva en 2030^8^.

La lactancia materna es considerada un fenómeno complejo y multifactorial^9^^,^^10^, en el que intervienen desde aspectos estructurales -como la creciente inserción de la mujer en el mercado laboral o las influencias culturales- hasta aspectos individuales como la experiencia previa de dificultades para amamantar^9^^-^^12^. Por ello, el proceso de inicio, instauración y mantenimiento de la lactancia materna se relaciona en ocasiones con una situación potencialmente ansiogénica y estresante, en el que algunas mujeres no alcanzan sus objetivos^13^^,^^14^. La carencia o ausencia de estrategias para enfrentar este proceso se ha asociado con ansiedad, perturbación emocional y tristeza crónica, pudiendo conducir a daños en el bienestar de la diada madre-hijo lactante^15^^,^^16^.

En esta perspectiva, resulta crucial que el equipo interprofesional encargado de liderar el abordaje y respaldo a la lactancia materna, conformado por matronas, enfermeras, pediatras y médicos de familia, esté capacitado para brindar un sólido respaldo a las personas lactantes mediante estrategias multicomponente durante todo. el periodo perinatal^17^^-^^19^. Estas estrategias incluyen la educación prenatal, la asesoría sobre lactancia materna en el posparto, el apoyo emocional -que abarca consuelo y aliento-, así como la planificación anticipada de visitas domiciliarias o en el centro asistencial^17^^-^^19^.

Dependiendo de sus recursos de afrontamiento, ante la adversidad algunas personas se enfrentan de manera más efectiva a los factores estresantes^20^. Conforme a la teoría salutogénica de Antonovsky^21^, las personas cuentan con recursos que pueden facilitar la gestión eficaz del estrés, definidos como recursos generales de resistencia (RGR). Son factores biológicos, materiales y psicosociales^22^ que facilitan a las personas percibir su vida como coherente, estructurada y comprensible. Los RGR clasifican en físicos; materiales, cognitivos y emocionales; valorativos; interpersonales y macro-socioculturales^22^. Una persona que tenga este tipo de recursos a su disposición o en su entorno inmediato tiene más oportunidades para hacer frente a los desafíos de la vida^20^. No obstante, más allá de la posesión de RGR, la clave radica en la capacidad de utilizarlos para construir experiencias coherentes que fomenten la salud, concepto conocido como el sentido de coherencia (SC)^21^. El SC de una persona consta de tres dimensiones: comprensibilidad (aspecto cognitivo), manejabilidad (aspecto conductual) y significatividad (aspecto motivacional). Cuanto más elevado sea el nivel de SC de las personas, más adecuadamente contenderán con los factores estresantes y, en consecuencia, mantendrán su salud^22^. El SC supone un constructo universal que puede hallarse en los humanos independientemente de su cultura, religión, género o clase social^23^.

Ante la premisa de que el SC puede actuar como moderador de las fuentes de estrés inherentes al entorno y como factor protector ante sucesos negativos de la vida^24^, y dado que la lactancia materna se identifica como un proceso heterogéneo y complejo que origina experiencias ambivalentes en las mujeres^25^^,^^26^, se considera relevante estudiar cómo influye el SC en mujeres lactantes. La producción en investigación en torno al tema ha priorizado el análisis de los conocimientos, percepciones y representaciones culturales que inciden en las mujeres para desalentar la práctica de la lactancia materna^26^^-^^28^. Sin embargo, no se han identificado revisiones que identifiquen qué es aquello que contribuye, favorece o genera salud en el contexto de la dicha práctica.

Por consiguiente, el presente estudio de revisión se ha establecido con el objetivo de analizar el sentido de coherencia en mujeres lactantes, identificar los recursos generales de resistencia que lo modulan, y determinar las intervenciones profesionales que lo promueven.

## MATERIAL Y MÉTODOS

El diseño del estudio corresponde a una revisión de alcance, tipo de revisión que permite resumir la evidencia e identificar lagunas de conocimiento o áreas poco investigadas^29^. Se emplearon como guía las pautas establecidas por *Preferred Reporting Items for Systematic Reviews and Meta Analysis extension for Scoping Reviews* (PRISMA-ScR)^30^.

Entre marzo y mayo de 2023 se llevaron a cabo búsquedas electrónicas en las siguientes bases de datos bibliográficas: PubMed, Cochrane Library, PsycINFO, ScienceDirect y CINAHL. La búsqueda se realizó mediante palabras clave basadas en los siguientes términos Medical Subject Header (MeSH)^31^: “*Sense of coherence*”; “*Breastfeeding*”. Para combinar estos términos, se empleó AND como operador booleano. También, se realizó una revisión manual de las referencias bibliográficas de los artículos seleccionados. Finalmente, se consultó literatura gris en *ProQuest Dissertations & Theses* y en el metabuscador *Google Scholar* (Tabla 1)*.*

**Tabla 1:** Estrategias de búsqueda

Bases de datos	Palabras clave /	Algoritmo	Artículos potenciales
	Descriptores	de búsqueda	
Pubmed (MEDLINE)	#1 breastfeeding [Title/Abstract]	(#1 OR #2)	10
https://pubmed.ncbi.nlm.nih.gov	#2 breastfeeding [MeSH Terms]	AND	
	#3 sense of coherence [Title/Abstract]	(#3 OR #4)	
	#4 sense of coherence [MeSH Terms]		
*Sciencie Direct*	#1 breastfeeding	(#1) AND (#2)	286
www.sciencedirect.com	#2 sense of coherence		
Cochrane *Library*	#1 breastfeeding: ti,ab,kw	(#1) AND (#2)	2
https://www.cochranelibrary.com	#2 sense of coherence: ti,ab,kw		
CINHAL	#1 breastfeeding: MH	(#1) AND (#2)	9
https://search.ebscohost.com	#2 sense of coherence: MH		
PsycINFO	#1 SU: (breastfeeding)	(#1) AND (#2)	1
http://search.proquest.com/psycinfo	#2 SU: (sense of coherence)		
Bases de datos *de literatura gris*			
*ProQuest Dissertations & Theses*	#1 SU: (breastfeeding)	(#1) AND (#2)	5
http://search.proquest.com/pqdt/dissertations	#2 SU: (sense of coherence)		
*Google Scholar*	#1 allintitle: “breastfeeding”	(#1) AND (#2)	3
https://scholar.google.es	#2 allintitle: “sense of coherence”		

ti: title word; ab: abstract word; kw: key words (MeSH and other); MH: CINAHL heading; SU: subjects; todas las búsquedas se realizaron el 11 de marzo de 2023.

Los criterios de inclusión fueron: estudios que analizaran el sentido de coherencia de las mujeres que lactan, artículos de fuentes primarias, escritos en inglés, español o portugués. Debido a la relevancia de los estudios publicados en 2011 en esta materia, decidimos incluir estudios publicados durante los últimos 12 años. Se excluyeron los artículos no realizados en humanos, presentaciones de carteles, ponencias en congresos, estudios de caso único, protocolos de estudio, y artículos de revisión.

Dos investigadores, de forma independiente, realizaron la búsqueda y clasificaron los estudios, según los criterios de elegibilidad, como incluido, excluido o incierto. Las discrepancias en la clasificación de los artículos se discutieron hasta alcanzar un consenso; se registraron los motivos de exclusión.

Las variables analizadas en esta revisión fueron:

- *Cualquier tipo de lactancia materna*, entendida como la toma del bebé de cualquier cantidad de leche materna con o sin otros líquidos o alimentos^32^;
- *Sentido de coherencia (SC) de las madres lactantes*, medido en los estudios cuantitativos incluidos en la revisión mediante el cuestionario SOC-13, escala testada psicométricamente en diversas muestras: personas con enfermedades crónicas^33^^-^^35^, con trastornos mentales^36^^,^^37^, y con problemas bucodentales^38^^-^^40^, que demuestra buena fiabilidad y validez transcultural, con valores de alfa (α) de Cronbach entre 0,70 y 0,92^41^. Este cuestionario consta de 13 ítems divididos en tres dimensiones: comprensibilidad, manejabilidad y significatividad. Cada ítem se puntúa con una escala tipo Likert entre 1 = muy a menudo y 7 = rara vez o nunca. El rango de puntuación total es de 13 a 91; cuanto mayor sea la puntuación, mayor será el SC^23^.

El primer autor extrajo los siguientes datos de cada artículo seleccionado: autor, año de publicación, lugar de estudio, objetivo del estudio, tipo y diseño del estudio, tamaño de muestra, variables analizadas, instrumentos, calidad documental evaluada a través de niveles de evidencia del Instituto Joana Briggs (JBI)^42^, y fiabilidad y validez (α de Cronbach).

Los resultados se presentaron de forma descriptiva agrupándose en tres objetivos de este estudio: el impacto del sentido de coherencia en las mujeres lactantes, los recursos generales de resistencia que lo modulan, y las intervenciones profesionales que lo promueven.

Los dos autores evaluaron la calidad metodológica de los estudios de forma independiente; las discrepancias se discutieron y resolvieron conjuntamente. Se utilizó la herramienta *Integrated quality criteria for review of multiple study designs* (ICROMS) para evaluar la calidad metodológica y el riesgo de sesgo de los artículos de cohortes y cualitativos, cuyas puntuaciones mínimas se establecieron en 18 y 16 puntos, respectivamente^43^. La evaluación de la calidad de los estudios transversales se realizó mediante la herramienta *Strengthening the reporting of observational studies in epidemiology* (STROBE), cuya puntuación de corte se estableció en 18 puntos^44^.

## RESULTADOS

**Figura 1:**
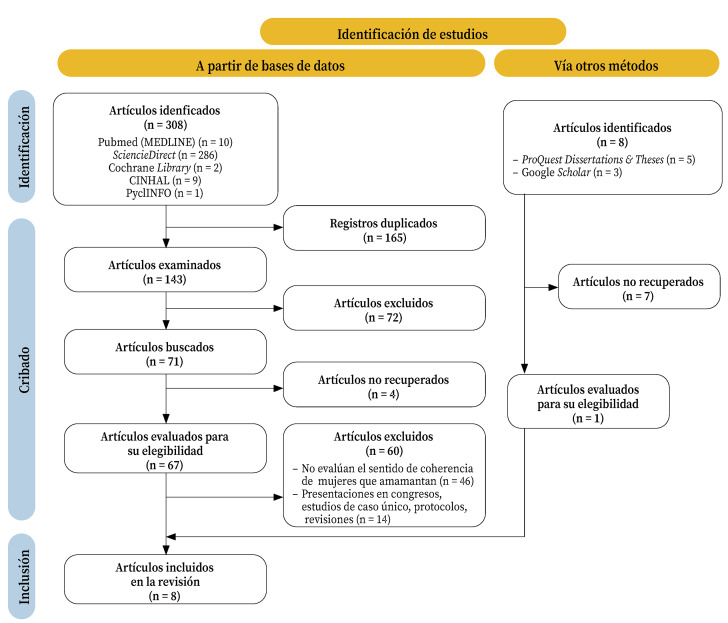
Diagrama de flujo que muestra el proceso de selección de estudios.

Los 316 estudios identificados se cribaron mediante la lectura de sus títulos y resúmenes, eliminado duplicados y aquellos artículos que no cumplían los criterios de selección. Finalmente, se incluyeron ocho artículos en la revisión de alcance (Figura 1).

### Características de la muestra

Los ocho estudios que conformaron la muestra^44^^-^^51^ correspondieron a investigaciones primarias, tres de ellos de naturaleza cualitativa (dos narrativos^45^^,^^46^ y uno etnográfico^47^) y cinco con enfoque cuantitativo (cuatro longitudinales prospectivos^48^^-^^51^ y uno transversal^44^). Los artículos provenían de seis países, el 40% de los países nórdicos (dos de Suecia^48^^,^^49^ y uno de Finlandia^45^). La revisión incluyó 890 madres lactantes y 26 profesionales; el tamaño muestral osciló entre 7^45^ y 324 mujeres lactantes^48^. El método de muestreo para captar a las madres en los estudios fue predominantemente por conveniencia, excepto dos investigaciones que emplearon muestreo por conglomerados^45^ y en bola de nieve^46^. En la Tabla 2 se presentan en detalle todas las características de los estudios incluidos en la revisión.

**Tabla 2:** Características de la metodología de los estudios revisados

Estudios cualitativos			
Autor /Año / País	Objetivo	Muestra / Áreas / Diseño	Fuentes de datos / Recopilación / Integridad
Byrom y col / 2021^47^ / Reino Unido	Explorar desde el SC la influencia de la iniciativa amiga del niño (UNICEF) en la cultura organizativa de un servicio de maternidad. Explorar las percepciones y experiencias de las madres lactantes de dicho servicio.	- n= 21 madres lactantes; - n= 26 profesionales (16 matronas); - Etnográfico	- Observación de participante y entrevistas; - Notas y reflexiones grabadas en audio y/o a través de un dictáfono para captar tanto el lenguaje verbal como no verbal; - Reflexividad; - Reuniones de supervisión para explorar el posicionamiento reflexivo; - Notas de campo revisadas por pares.
Kolanen y col / 2016^45^ / Finlandia	Examinar las percepciones de las mujeres somalíes sobre la lactancia materna utilizando un enfoque salutogénico	- n= 7 madres lactantes somalíes residentes en Finlandia; - Rural; - Narrativo	- Entrevistas en grupos focales; - Entrevistas grabadas por audio y transcritas palabra por palabra; - Reflexividad; - Comprobación de los informantes clave del análisis inicial.
Thomson y Dykes / 2011^46^ / Reino Unido	Proporcionar una interpretación teórica de la ‘comprensibilidad’, ‘manejabilidad’ y ‘significado’ de las experiencias de lactancia de las mujeres	- n= 25 madres lactantes; - Urbana, suburbana y rural; - Narrativo	- Grupos focales y entrevistas individuales; - Entrevistas grabadas digitalmente y transcritas palabra por palabra; - Comprobación de los participantes del análisis inicial; - Análisis y recopilación de los datos simultáneo con software MaxQDA; - Reflexión de los investigadores de los datos auditados
*Estudios cuantitativos*			
*Autor /Año / País*	*Objetivo*	*Muestra / Áreas / Diseño*	*Instrumentos / Fiabilidad y validez*
Grandberg y col / 2020^48^ / Suecia	Investigar factores asociados con el disfrute de la LM por madres primerizas, y la duración de la LM	- n= 324 madres lactantes; - primera semana del nacimiento hasta 2 años; - Urbana, suburbana, rural; - Observacional, prospectivo, de cohortes	- SOC-13, MIRF, QDR36, cuestionarios estructurados; - α=0,86 en primera semana posparto, α=0,896 a los seis meses, α=0,91 al año y a los dos años
Linden y col / 2018^49^ / Suecia	Explorar e investigar las asociaciones entre la LM, el bienestar y el control de la DM1	- n= 125 madres lactantes con DM1; - Hasta 6 meses postparto; - Urbana y suburbana; - Observacional, prospectivo, de cohortes	- SOC-13, W-BQ12, SWEDES-10, cuestionarios estructurados; - Cuestionario test-retest y correlaciones ítem-puntuación total; - α=0,74 - 0,96
Nakarani y col / 2020^51^ / Japón	Evaluar la autoeficacia en la LM de madres con bebés en UCIN e identificar factores relacionados con la autoeficacia	- n= 198 madres lactantes con bebes ingresados en UCIN; - 3-7 días hasta 1 mes tras iniciar la lactancia; - Urbana y suburbana; - Observacional, prospectivo, de cohortes	- EPDS (versión japonesa), BSES-SF, PIMQ, SOC-13; - α=0,76 a los 3-7 días tras iniciar la lactancia, α=0,83 un mes tras iniciar la lactancia
Pavicic Bosnjak y col / 2012^50^ / Croacia	Traducir y evaluar psicométricamente el BSES-SF entre mujeres lactantes en Croacia	- n= 190 madres lactantes; - Hasta los 6 meses; - Urbana y suburbana; - Observacional, prospectivo, de cohortes	- SOC-13, BSES-SF, cuestionarios estructurados; - α=0,86

α: alfa de Cronbach; DM1: diabetes mellitus tipo I; LM: lactancia materna; SC: sentido de coherencia; UCIN la unidad de cuidados intensivos neonatales. Cuestionarios: BSES-SF: *Breastfeeding Self-Efficacy Scale-Short Form*; EPDS: *Edinburgh Postpartum Depression Scale*; MIRF: *Mother to Infant Relations and Feelings scale*; PIMQ: *Perception of Insufficient Milk Questionnaire*; QDR36: *Quality of the Couple’s Relationship scale*; SOC-13: *Sense Of Coherence scale*; SWEDES-10: *Swedish Diabetes Empowerment Scale 10*; W-BQ12: *short-form 12-item Well-Being Questionnaire*.

Cuestionarios. BSES-SF: escala abreviada de autoeficacia de la lactancia materna; MIRF: escala de sentimientos y relaciones de madre a hijo; PIMQ: cuestionario de leche insuficiente percibida; QDR36: calidad de la relación de pareja; SOC-13: escala de sentido de coherencia; SWEDES-10: escala sueca de empoderamiento de la diabetes; W-BQ12: cuestionario de bienestar de 12 ítems.

Los estudios prospectivos de cohorte longitudinal obtuvieron un nivel de evidencia 3c^47^^-^^49^, el estudio observacional transversal un nivel 4b^45^, y las investigaciones cualitativas 5c^46^^,^^50^^,^^51^. Todos los estudios obtuvieron un grado de recomendación B.

### Impacto del sentido de coherencia en las mujeres lactantes

Los estudios muestran una asociación positiva entre un nivel elevado de SC materno y una mayor duración de la lactancia^45^^,^^47^^-^^51^ y una mayor autoeficacia de la LM^49^^,^^50^^,^^52^. Además se correlacionó con un mayor disfrute de la experiencia de amamantar (satisfacción con la lactancia materna)^47^^,^^50^, un estado de bienestar adecuado^48^^,^^50^^,^^51^ y un mayor vínculo o apego de la diada madre-lactante^46^^,^^50^ (Tabla 3).

**Tabla 3:** Descripción de los principales hallazgos según objetivos

Impacto del SC en mujeres lactantes	
^45^Cortelo y col^44^ / 2018	- Las madres con menor SC tenían más probabilidades de destetar prematuramente (p<0,01)
	- Las madres con mayor SC tenían 1,82 veces más probabilidades de mantener la lactancia materna por períodos más prolongados (p=0,02)
Granberg y col^48^ / 2020	El SC correlacionó positivamente con:
	- El disfrute de la LM a la semana del nacimiento (r_s_=0,263; p<0,001) y a los seis meses (r_s_=0,233; p=0,007)
	- La duración de la lactancia (r_s_=0,241; p=0,005)
	- La calidad de la relación madre-hijo en la primera semana postparto (r_s_=0,445; p=0,026), a los seis meses (r_s_=0,424; p=0,035) y al año (r_s_=0,527, p≤ 0,001)
Linden y col^49^ / 2018	- El grado de SC de las madres correlacionó positivamente con el bienestar general (r_s_=0,25, p=0,01)
	- El grado de SC correlacionó negativamente con la necesidad de apoyo profesional para controlar su diabetes
Pavicic Bosnjak y col^50^ / 2012	- Una mayor autoeficacia en la lactancia se asoció a un SC fuerte a nivel general (r_s_=0,35, p<0,001) y en las subescalas de comprensión (r_s_=0,35, p = 0,001), manejabilidad (r_s_=0,26, p<0,001) y significado (r_s_=0,20, p=0,005)
	- Un alto SC al alta del hospital se asoció a LM exclusiva a los 6 meses (p=<0,001)
Nakatani y col^51^ / 2020	- Un SC más alto al mes tras el inicio de la lactancia de los lactantes hospitalizados en la UCIN predijo una mayor autoeficacia de la LM (p=0,029)
Thomson y Dykes^46^ / 2011	- Las mujeres que perseveraron y mantuvieron la LM fueron las que mantuvieran un SC fuerte
	- El SC fuerte se asoció a tener una mayor confianza o autoeficacia, mayor disfrute de su experiencia y al establecimiento de un vínculo sólido con su hijo
*RGR que promueven el SC*	
Cortelo y col^44^ / 2018	- Disponer de un nivel económico y educativo medio-alto; edad materna mayor de 30 años; poseer estrategias de afrontamiento cognitivas y afectivas de afrontamiento (p=<0,0001)
	- Percibir buen apoyo por parte de miembros de la familia, entorno social y pareja (ns)
	- Experiencia previa de LM
	- Las madres que obtuvieron una puntuación mayor de 48 en la escala SOC-13 y por lo tanto presentaban niveles más elevados de SC, tenían más de 30 años y un nivel socio-económico medio-alto
Granberg y col^48^ / 2020	- Percibir una relación conyugal sólida correlacionó con tener buen nivel de SC (r_s_=0,240, p=0,002)
	- Experiencia previa en LM
Linden y col^49^ / 2018	- Buen estado y manejo de la salud
Kolanen y col^45^ / 2016	- La disponibilidad de fuentes de conocimiento y apoyo por parte de madres, mujeres somalíes con experiencia y profesionales sanitarios favorecieron el SC de las madres lactantes
	- El apoyo por parte sus parejas fue un aspecto clave para prevenir el abandono prematuro de la lactancia
	- Las creencias religiosas y culturales favorecieron que las mujeres completaran dos años de LM
Thomson y Dykes^46^ / 2011	- Disponer de apoyo sólido y cercano de profesionales sanitarios, amigos y familiares, sobre todo sus parejas y madres
	- Los grupos de apoyo entre pares favorecen la comprensibilidad y manejabilidad
	- Usar estrategias cognitivas, afectivas e instrumentales, y mostrar una actitud positiva facilitaban el afrontamiento y el desarrollo de la comprensibilidad, manejabilidad y significado
*Intervenciones que favorecen el SC*	
Byrom y col^47^ / 2021	- Las tareas burocráticas y rutinarias que se realizan en las salas de maternidad suponen barreras para ofrecer un buen cuidado y apoyo la LM, por causar falta de tiempo y disminuir la presencia y contacto de las matronas con las mujeres lactantes
	- Ofrecer intervenciones por parte de matronas, asesoras de lactancia y otros profesionales, basadas en suministrar apoyo práctico, informativo y emocional, favorecieron la comprensibilidad, manejabilidad y significatividad, respectivamente
Kolanen y col^45^ / 2016	- Comprensibilidad: que la información que se diese por parte de los profesionales sanitarios fuera concordante con la que ofertaban las madres de las mujeres lactantes somalíes y, a su vez, que aportase información sobre prácticas para la prevención de infecciones, posturas, el contacto piel con piel
	- Manejabilidad: interfirió el tipo de parto y la práctica de contacto piel con piel, así como el modelo centrado en la familia, dando un papel más activo al cónyuge
Thomson y Dykes^46^ / 2011	- Comprensibilidad: ofrecer información no tecnificada, práctica, consistente y concordante entre los profesionales y familiares, especialmente la pareja y sus madres
	- Manejabilidad: mostrar una posición terapéutica accesible y empática, dedicar tiempo y ofrecer una buena experiencia de nacimiento con el contacto piel con piel como comienzo. Favorecer grupos de apoyo entre pares
	- Significatividad: reforzar sus logros y afrontamiento de adversidades, así como no ejercer un clima de presión

LM: lactancia materna; ns: no significativo; RGR: recursos generales de resistencia; r_s_: coeficiente de correlación de Spearman (no paramétrico); SC: sentido de coherencia; SOC-13: escala de sentido de coherencia; UCIN la unidad de cuidados intensivos neonatales.

### Recursos generales de resistencia que modulan el sentido de coherencia

Se identificaron como RGR físicos, la obtención de una puntuación mayor o igual a 48 en la escala SOC-13, tener más de 30 años, poseer un buen estado y manejo de la salud^45^^,^^48^, y como recursos materiales ostentar un nivel socio-económico medio-alto (>4 salarios mínimos)^45^. Además, la experiencia previa en lactancia materna satisfactoria^45^^,^^46^^,^^50^ se identificó como un RGR cognitivo muy importante.

Disponer de un buen sistema de apoyo a la lactancia materna por parte de profesionales de la salud^46^^,^^48^^,^^50^^,^^51^, familiares, especialmente la pareja^45^^-^^47^^,^^50^ y la madre^45^^,^^46^^,^^50^, y laicos, como las madres de los grupos de apoyo entre pares a la lactancia materna^50^, fueron RGR interpersonales clave en el proceso de amamantamiento.

Los RGR valorativos destacados por las mujeres lactantes fueron poseer una actitud positiva por parte de las madres^45^^,^^47^^,^^50^ y disponer de sólidas estrategias cognitivas y afectivas de afrontamiento^45^^,^^50^.

Las creencias religiosas y culturales (por ejemplo, las mujeres somalíes que contemplan dos años de lactancia porque el Corán aboga por ello y porque su cultura enfatiza los beneficios de la lactancia) son RGR macro-socioculturales importantes^46^ (Tabla 3).

### Intervenciones profesionales promueven el sentido de coherencia

Las intervenciones profesionales que se han asociado con una mejora de la comprensibilidad de las mujeres lactantes son: dar información de manera práctica, individualizada y adaptada a cada caso, mostrar una actitud cercana^46^^,^^50^^,^^51^, ofrecer atención directa cara a cara, implicar a la pareja y a la madre en el proceso de lactancia, de manera que se favorezca que la información que fluya hacia las mujeres lactantes sea consistente y concordante por parte de todos^46^^,^^50^^,^^51^.

Las intervenciones que facilitan la manejabilidad de las mujeres con la lactancia materna son: procurar una experiencia de parto basada en el respeto y la evidencia, que asegure el contacto piel con piel directo, inmediato e ininterrumpido^46^^,^^50^; abogar por un enfoque terapéutico basado en la atención centrada en la familia de carácter accesible, continuo, personalizado, cercano y presencial^46^^,^^50^^,^^51^; mostrar siempre una actitud empática y sensible con la madre lactante^50^^,^^51^; enfatizar en la visita domiciliaria postparto la consejería en lactancia^46^; y formar o propiciar grupos entre pares^50^.

En relación a las intervenciones que promueven la significatividad materna de amamantar se han detectado: propiciar apoyo afectivo y escucha activa, permitiendo que las madres verbalicen sus problemas experimentados durante el proceso de amamantar^50^^,^^51^; transmitir tranquilidad, reforzando sus logros o valorando positivamente cómo afrontan las dificultades o problemas con el amamantamiento; y hablarles sobre los beneficios infantiles, materno, sociales y económicos de la lactancia materna a corto y largo plazo, así como de los riesgos de no amamantar^50^^,^^51^.

**Figura 2:**
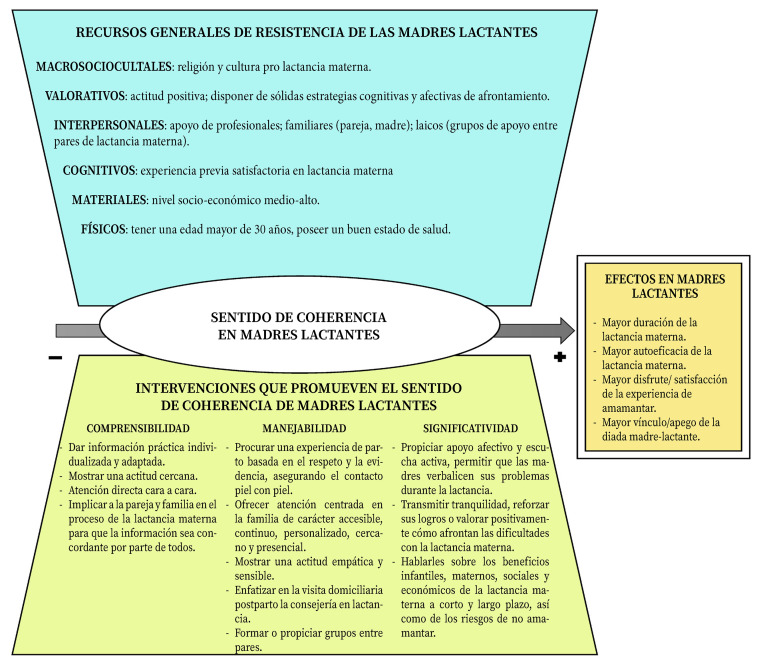
Representación gráfica de los resultados.

La Figura 2 sintetiza los resultados detectados en esta revisión de alcance.

### Evaluación de la calidad metodológica

Todos los estudios de cohortes superaron la puntuación mínima de calidad global mediante la herramienta ICROMS^43^, obteniendo puntuaciones entre 20 y 23. Cumplieron los criterios obligatorios de poseer una declaración clara de los objetivos de la investigación; de manejo del sesgo entre grupos; manejo del sesgo de la comparabilidad de los resultados, y manejo del sesgo de seguimiento abordando datos de resultados incompletos (Tabla 4). También los estudios cualitativos superaron la puntuación mínima con ICROMS^43^, obteniendo puntuaciones entre 17 y 18. Cumplieron los criterios obligatorios de poseer una declaración clara de los objetivos de la investigación; una selección del diseño del estudio adecuado, y manejo del sesgo de muestreo y reclutamiento (Tabla 4).

**Tabla 4:** Evaluación de la calidad de los estudios seleccionados mediante ICROMS

Estudio		Dimensiones ICROMS*							Puntuación Total
		1.	2.	3.	4.	5.	6.	7.	
*Diseño cualitativo*	Nº de criterios por dimensión	*3*	*1*	*1*	*1*	*1*	*1*	*5*	
Byrom y col, 2021 ^47^		5	2	1	2	2	1	5	18
Kolanen y col, 2016 ^45^		5	2	1	2	2	1	4	17
Thomson y Dykes, 2011 ^46^		6	2	1	1	1	1	5	17
*Diseño de cohortes*	Nº de criterios por dimensión	*1*	*1*	*3*	*1*	*1*	*1*	*5*	
Grandberg y col, 2020^48^		2	2	5	2	1	1	7	20
Linden y col, 2018^49^		2	2	6	2	2	1	8	23
Nakarani y col, 2020^51^		2	2	5	2	1	0	8	21
Pavicic Bosnjak y col, 2012^50^		2	2	6	2	2	1	7	22

ICROMS: *integrated quality criteria for review of multiple study designs;* Dimensiones: 1. Objetivos y justificación, 2. Muestreo, 3. Medidas de resultado, 4. Hacer un seguimiento, 5. Otros aspectos del estudio, 6. Rigor analítico, 7. Otra consideración; Puntuación: 2 = criterio cumplido; 1 = poco claro; 0 = criterio no cumplido.

El estudio transversal obtuvo una puntuación de calidad metodológica global de 19/22 mediante la herramienta STROBE^44^. Se restó puntuación por no describir todas las medidas adoptadas para afrontar fuentes potenciales de sesgo, por no describir otros análisis efectuados, y por la dificultad para generalizar los resultados (Tabla 5).

El nivel de acuerdo entre los dos evaluadores fue del 94%.

**Tabla 5:** Evaluación de la calidad del estudio transversal (Cortelo y col 2018^44^) mediante la herramienta STROBE

Criterios STROBE	Puntación	Criterios STROBE	Puntación
1. Título y resumen	1	12. Métodos estadísticos	1
2. Contexto/fundamentos	1	13. Participantes	1
3. Objetivos	1	14. Datos descriptivos	1
4. Diseño del estudio	1	15. Datos de las variables de resultado	1
5. Contexto	1	16. Resultados principales	1
6. Participantes	1	17. Otros análisis	0
7. Variables	1	18. Resultados clave	1
8. Fuentes de datos/medidas	1	19. Limitaciones	1
9. Sesgos	0	20. Interpretación	1
10. Tamaño muestral	1	21. Generabilidad	0
11. Variables cuantitativas	1	22. Financiación	1
Total			19

STROBE: *strengthening the reporting of observational studies in epidemiology*; Puntuación: 1= presencia del indicador de calidad, 0 = ausencia.

## DISCUSIÓN

Esta revisión se fundamentó en analizar el impacto del SC en mujeres lactantes, establecer los recursos generales de resistencia que lo modulan, así como determinar las intervenciones que lo promueven. Hasta donde sabemos, esta es la primera revisión de alcance que analiza el SC y determina qué factores contribuyen, favorecen o generan salud en el contexto de la práctica de la LM. Debido a la escasez de literatura, no fue posible determinar con exactitud el impacto del SC sobre la experiencia de LM; no obstante, los resultados convergen en la relevancia de considerar esta variable en la práctica clínica.

Los estudios han identificado que un SC más alto en las madres contribuyó en la autoeficacia del amamantamiento^49^^,^^50^^,^^52^. Esto se traduciría en que puedan ser más competentes, aplicando el conocimiento, la experiencia y las habilidades relacionadas con la práctica. Además de aprovechar el apoyo brindado por amigos y familiares para configurar o mejorar su confianza en la LM y hacer frente a los desafíos que conlleva la maternidad. Todo ello puede repercutir en su bienestar, originar que sea más favorable poder disfrutar de la vivencia, generar experiencias más duraderas de LM y por lo tanto propiciar la diada madre-lactante. No obstante, los artículos de la muestra han explorado el SC materno hasta los dos años posparto, por lo que se precisan estudios que evalúen la trayectoria del SC desde el embarazo hasta más allá de los dos años.

Desde una perspectiva salutogénica basada en los componentes que conforman el SC^50^^,^^51^, se podría decir que aquellas madres con SC alto tienen un mayor nivel de *comprensibilidad,* lo que se relacionaría con la decisión activa y consciente de la mujer de amamantar; mayor *manejabilidad,* que se asociaría con la confianza de las madres en su capacidad para enfrentar las dificultades de la LM, utilizando los recursos generales de resistencia disponibles; y mayor *significatividad*, enlazado con poseer un propósito, por lo que, incluso ante los obstáculos, se opta por continuar con la lactancia.

Los RGR fueron piedras angulares en el desarrollo de un elevado sentido de coherencia en las madres lactantes y están reconocidos como buenas prácticas en salud^53^^-^^55^. Los RGR hallados en este estudio estaban enmarcados dentro de las seis categorías de la clasificación de Antonovsky^22^. Se destacaron en el contexto de la lactancia materna especialmente los RGR interpersonales, pues el apoyo socioemocional por parte de profesionales, familiares y en especial de la pareja, fue uno de los RGR cruciales en este estudio. Esto coincide con que la mayoría de las madres que encuentran apoyo en su entorno para amamantar, viven una experiencia de lactancia más gratificante, enriquecedora, extensa en el tiempo y más saludable para ellas y sus hijos^56^. Además, aquellas madres con un SC más elevado son proclives a mantener relaciones satisfactorias con su pareja y familia^57^. Esto remarca la relevancia de incluir a la pareja y familiares en la relación terapéutica profesional. Por el contrario, los comentarios negativos de las personas allegadas, a pesar de que no tienen tanta influencia en la decisión de amamantar, sí que perjudican notablemente la manejabilidad y comprensibilidad del SC^50^.

También cobraron importancia en esta revisión los RGR valorativos, como la posesión de estrategias de afrontamiento cognitivas y afectivas, las cuales favorecían un SC alto y la preservación de la LM. En el estudio de Libera y col^58^ se determinó que las madres con un SC elevado poseían más tendencia a adoptar estrategias relacionadas con la búsqueda de apoyo y actividades sociales; por el contrario las que tenían el SC más bajo, emplearon estrategias centradas en las emociones^58^. De hecho, el puerperio y la instauración de la lactancia suponen procesos que exigen notables cambios fisiológicos, emocionales y sociales, que demandan adecuadas adaptaciones^59^^,^^60^. Sin embargo, en el ámbito de la LM, existe poca información que profundice y especifique qué tipo de perspectiva estratégica adoptan las madres lactantes para potenciar el SC y por lo tanto su experiencia de LM^50^.

Poseer una actitud positiva también fue un RGR valorativo clave. Las mujeres con actitudes positivas, independientemente de la intervención profesional, mostraban tasas más altas de iniciación y mantenimiento de la lactancia materna exclusiva hasta los seis meses^61^. También se identificó la experiencia previa exitosa de LM como un RGR cognitivo, en concordancia con otras investigaciones que relacionaron este factor con una lactancia materna exclusiva a los seis meses ^(^^62^^,^^63^.

Las intervenciones profesionales identificadas que promueven el SC en las mujeres lactantes son un componente esencial para cumplir con la responsabilidad de salud pública de aumentar las tasas de lactancia materna^64^. Se ha visualizado que son útiles tanto para fomentar la práctica de LM como para favorecer la adopción del rol y la diada entre la madre-lactante^65^. Asimismo, es factible que sean efectuadas por matronas, enfermeras^66^ y otro personal sanitario implicado en el abordaje de la lactancia materna^67^; este último fue considerado una fuente importante de apoyo para las familias que amamantan, relacionándose sus actuaciones con la contribución en la mejora de las tasas de LM^68^.

Varias investigaciones incluidas en esta revisión concordaron en que la vivencia del parto y la práctica del contacto piel con piel influía en el nivel de SC en las mujeres lactantes, en línea con la evidencia de que, más que el modo de nacimiento, es el momento de contacto e instauración de la primera alimentación el determinante clave para promover la LM^69^^-^^71^. Además la literatura ha reportado que el ser madre y tener un nivel bajo de SC, supone un factor de riesgo para tener un parto prematuro^72^, finalizar con una cesárea^73^, o tener complicaciones en el parto^74^. En esta línea, sería sugerente explorar el SC en mujeres que lactan, tras embarazos por técnicas de reproducción asistida, pues se ha documentado^75^, que aquellas que consiguen ser madres tras ser sometidas a técnicas de reproducción asistida, reaccionan con un aumento significativo del SC al año de seguimiento.

Las intervenciones basadas principalmente en el apoyo cara a cara y centradas en la familia tuvieron más probabilidades de éxito con las mujeres lactantes, lo que coincide con otros estudios^19^. Sin embargo, prácticas como administrar folletos informativos con información técnica, esperar a que las mujeres se los lean y hagan preguntas, y mostrar una posición terapéutica de experto^50^, suponen una barrera para alcanzar la comprensibilidad y que, finalmente, las madres se den por vencidas ante las dificultades de la lactancia. Ante la evolución de las tecnologías de comunicación, sería interesante estudiar intervenciones para fortalecer el SC y favorecer la lactancia a través de las redes sociales^64^.

Este estudio destaca las intervenciones basadas en grupos de apoyo entre pares. Cuando el apoyo entre pares es dirigido por un profesional, tiene un mayor efecto en el inicio, mantenimiento y duración de la lactancia materna^61^, además de tener un mayor impacto que el apoyo individual^76^, pues resulta una práctica que cultiva la autoestima y confianza de las mujeres en el proceso de amantar, además de reducir el aislamiento social^77^.

Para promover el SC de las madres lactantes, sería relevante que matronas y enfermeras se esfuercen por fortalecer dicha cualidad desde el período fértil (pues se ha demostrado que el SC disminuye durante los primeros meses después del parto^77^) y garantizando la continuidad asistencial durante el postparto. Será importante considerar la familia como unidad de cuidado y a las mujeres que lactan como personas con sentimientos, experiencias y metas genuinas. Enfatizar la detección precoz de las mujeres con bajo SC permitirá propiciar intervenciones tempranas que prevengan el destete precoz y generen prósperas experiencias de lactancia materna.

Entre las limitaciones de esta revisión, destacan la dificultad de generalización de los resultados, ya que los artículos que la conformaron presentaron un nivel de evidencia y grado de recomendación bajo.

Los resultados de esta revisión apuntan a que un nivel elevado de SC materno durante el posparto se asocia con mayor duración de la LM, autoeficacia, disfrute de la experiencia de amamantar, bienestar adecuado y el fomento de la diada madre-lactante. Entre los RGR que pueden ayudar a las mujeres a percibir sus lactancias maternas como coherentes, se destaca especialmente el apoyo percibido por sus parejas y madres, poseer una experiencia previa en amamantar, el uso de estrategias cognitivas y afectivas y mostrar una actitud positiva. Desarrollar intervenciones profesionales centradas en la familia, caracterizadas por la continuidad, integralidad y basadas en buenas prácticas en LM, será fundamental para mejorar el SC de las madres lactantes y así promocionar la lactancia.
